# Whole genome investigation of a divergent clade of the pathogen *Streptococcus suis*

**DOI:** 10.3389/fmicb.2015.01191

**Published:** 2015-11-04

**Authors:** Abiyad Baig, Lucy A. Weinert, Sarah E. Peters, Kate J. Howell, Roy R. Chaudhuri, Jinhong Wang, Matthew T. G. Holden, Julian Parkhill, Paul R. Langford, Andrew N. Rycroft, Brendan W. Wren, Alexander W. Tucker, Duncan J. Maskell

**Affiliations:** ^1^Department of Veterinary Medicine, University of CambridgeCambridge, UK; ^2^Department of Paediatrics, University of CambridgeCambridge, UK; ^3^Department of Molecular Biology and Biotechnology, University of SheffieldSheffield, UK; ^4^School of Medicine, University of St AndrewsSt Andrews, UK; ^5^The Wellcome Trust Sanger Institute, Wellcome Trust Genome CampusCambridge, UK; ^6^Section of Paediatrics, Department of Medicine, Imperial College LondonLondon, UK; ^7^Royal Veterinary CollegeHatfield, UK; ^8^Faculty of Infectious and Tropical Diseases, London School of Hygiene & Tropical MedicineLondon, UK

**Keywords:** *Streptococcus suis*, divergent, genome, phylogeny, MLST, virulence, capsule, recombination

## Abstract

*Streptococcus suis* is a major porcine and zoonotic pathogen responsible for significant economic losses in the pig industry and an increasing number of human cases. Multiple isolates of *S. suis* show marked genomic diversity. Here, we report the analysis of whole genome sequences of nine pig isolates that caused disease typical of *S. suis* and had phenotypic characteristics of *S. suis*, but their genomes were divergent from those of many other *S. suis* isolates. Comparison of protein sequences predicted from divergent genomes with those from normal *S. suis* reduced the size of core genome from 793 to only 397 genes. Divergence was clear if phylogenetic analysis was performed on reduced core genes and MLST alleles. Phylogenies based on certain other genes (*16S rRNA, sodA, recN*, and *cpn60*) did not show divergence for all isolates, suggesting recombination between some divergent isolates with normal *S. suis* for these genes. Indeed, there is evidence of recent recombination between the divergent and normal *S. suis* genomes for 249 of 397 core genes. In addition, phylogenetic analysis based on the *16S rRNA* gene and 132 genes that were conserved between the divergent isolates and representatives of the broader *Streptococcus* genus showed that divergent isolates were more closely related to *S. suis*. Six out of nine divergent isolates possessed a *S. suis*-like capsule region with variation in capsular gene sequences but the remaining three did not have a discrete capsule locus. The majority (40/70), of virulence-associated genes in normal *S. suis* were present in the divergent genomes. Overall, the divergent isolates extend the current diversity of *S. suis* species but the phenotypic similarities and the large amount of gene exchange with normal *S. suis* gives insufficient evidence to assign these isolates to a new species or subspecies. Further, sampling and whole genome analysis of more isolates is warranted to understand the diversity of the species.

## Introduction

*Streptococcus suis* is a Gram positive pathogen of pigs that causes septicemia, meningitis and arthritis, amongst other clinical manifestations, posing a substantial burden on the pig industry (Gottschalk et al., 2007). It is also an important emerging zoonotic pathogen in humans (Gottschalk et al., 2010). Generally, human cases are reported in people who have had close contact with pigs and related products (Gottschalk et al., 2007). There have been two *S. suis* epidemics reported in humans, both in China, one in 1998 and one in 2005, and the pathogen is the principal cause of adult bacterial meningitis in Vietnam and other South East Asian countries (Wertheim et al., 2009; Gottschalk et al., 2010).

In the laboratory, isolates are identified as being *S. suis* by a series of biochemical tests. Four initial characteristics indicative of *S. suis* include an inability to grow on agar containing 6.5% NaCl, the production of acid when grown in trehalose or salicin broths and, importantly, a negative Voges-Proskauer (VP) test for acetoin production (Higgins et al., 1990). Additional discriminatory biochemical tests for the identification of *S. suis* include arginine dihydrolase activity, the production of acid from the breakdown of lactose, sucrose, or inulin and the inability to ferment glycerol, mannitol, and sorbitol (Higgins and Gottschalk, 1990). Once an isolate is identified as *S. suis*, it can be further characterized by serotyping based on the capsular antigen. These biochemical tests are the primary means of identification of *S. suis*, but to detect diversity within *S. suis* populations more discriminatory molecular typing methods are required. Molecular typing using the *16S rRNA* and chaperonin 60 (*cpn60*) gene sequences indicates that the *S. suis* populations are genetically diverse (Chatellier et al., 1998; Hill et al., 2005), but the *16S rRNA* gene sequences from most of the serotype reference isolates were between 97 and 100% identical. The exceptions to this high degree of conservation were the genes from the reference isolates for serotypes 32, 33, and 34 where the identity dropped to as low as 93.94% compared to the other serotypes (Chatellier et al., 1998). Further investigation reclassified serotypes 32 and 34 as being more closely related to *Streptococcus orisratti* based on the *16S rRNA* and *cpn60* gene sequences (Brousseau et al., 2001; Hill et al., 2005). *S. orisratti*, was originally isolated from the oral cavity of Sprague-Dawley rats, with no evidence of colonization or disease association in pigs (Zhu et al., 2000), although serotypes 32 and 34 continued to be isolated from diseased pigs in Canada and China (Messier et al., 2008; Wang et al., 2012; Gottschalk et al., 2013). The sequences of other housekeeping genes, including the gene encoding manganese-dependent superoxide dismutase (*sodA*; Glazunova et al., 2009) and a recombination/repair gene (*recN*) have revealed variation between *S. suis* isolates (Glazunova et al., 2010; Le et al., 2013; Ishida et al., 2014). It has been suggested that the reference isolates of serotypes 20, 22, 26, and 33 (hereafter referred to as “divergent” *S. suis*) are only distantly related to *S. suis* based on DNA–DNA hybridization and phylogenies of the sequences of *sodA* and *recN* (Le et al., 2013; Ishida et al., 2014). Only recently, the reference isolates of serotypes 20, 22, and 26 were proposed to form a new species, *Streptococcus parasuis*. sp. *nov*, based on the diversity of *recN* gene sequence and the average nucleotide identity values of their whole genome sequences compared to the genome sequences of recognized isolates of *S. suis* (Nomoto et al., 2015). Other than this study, all previous studies reporting divergence in *S. suis* focused on the serotype reference isolates and used a maximum of just two genes. An improvement on single gene methods is the availability of a multilocus sequence typing (MLST) scheme that describes variation of the housekeeping genes (*aroA, cpn60, dpr, gki, mutS, recA*, and *thrA;* King et al., 2002). However, even with MLST, diversity is only based on a small proportion of the genome (seven genes). DNA–DNA hybridization methods have been limited to comparisons of the reference isolates (Le et al., 2013).

Here, we report an analysis of the whole genome sequences of nine divergent isolates of *S. suis*. These isolates have phenotypic characteristics, such as disease profile, biochemical profile and serotype of *S. suis*, and can recombine within the *S. suis* population but their genome sequences are highly divergent from those of *S. suis*. Our research provides the basis for a more comprehensive taxonomic classification of the *S. suis* species by use of whole genome sequences, highlighting the fact that there is still a wealth of diversity of this significant zoonotic pathogen of pigs, that needs to be explored.

## Materials and Methods

### Bacterial Isolation, Serotyping, and Characterization

Field isolates of *S. suis* from the UK were from tissues of pigs submitted from farms in England and Wales during 2010 and 2011 to the Animal Health and Veterinary Laboratories Agency (AHVLA), now part of the Animal and Plant Health Agency (APHA). This resulted in a collection of 184 isolates. A further 153 isolates were from human clinical cases of meningitis in Vietnam and 32 isolates were from tissues obtained from healthy slaughterhouse pigs in Vietnam. Six additional isolates were from clinical pig cases in Vietnam (Weinert et al., 2015). The methods for isolation, biochemical characterization, serotyping, phenotypic grouping, and genome sequencing and assembly of these *S. suis* isolates is detailed in Weinert et al. (2015). Fifteen publically available genome sequences of *S. suis* were also included in the analysis presented here, giving a total of 390 isolates of *S. suis* in this comparative study. The phenotypic profiles of all *S. suis* isolates used in this study are listed in the supplementary material (Supplementary Table S1). These isolates are referred to as normal *S. suis* in this study to differentiate them from the divergent isolates of *S. suis*.

The collection of nine isolates of *S. suis* analyzed as being divergent in this study is shown in **Table 1**. All isolates are referred to by their ID number. Six of these isolates (**Table 1**) were collected from pigs in the UK as part of the collection of 184 isolates described above (Supplementary Table S1). S003 and S007 were pig clinical isolates associated with respiratory and systemic-brain infection respectively. LS6, LS7, LS17, and LS19 were non-clinical isolates. Previously published genome sequences from three divergent Canadian isolates of *S. suis* were also included in this study (**Table 1**). These were clinical isolates but there was no information available regarding the site of isolation from within the diseased animal (Chen et al., 2013). The European National Archive (ENA) accession numbers for the divergent genomes are given in **Table 1** and for the normal *S. suis* genomes used in study are in Supplementary Table S1.

**Table 1:** Phenotypic characteristics of the divergent isolates.

Divergent *Streptococcus suis* isolate	ID	Disease association (site of isolation in parentheses)	Serotype	Host	Country	Source	Accession number
SS007	S007	Clinical (systemic-brain)	4	Pig	UK	This study	ERS132358
SS1003	S003	Clinical (respiratory)	22	Pig	UK	This study	ERS132537
LSS17	LS17	Non-clinical	NT	Pig	UK	This study	ERS132379
LSS19	LS19	Non-clinical	4	Pig	UK	This study	ERS132381
LSS6	LS6	Non-clinical	NT	Pig	UK	This study	ERS132368
LSS7	LS7	Non-clinical	NT	Pig	UK	This study	ERS132369
86-5192	5192	Clinical (NK)	20	Calf	Canada	Chen et al., 2013	DRR018567
89-4109	4109	Clinical (NK)	26	Pig	Canada	Chen et al., 2013	DRR018569
88-1861	S22	Clinical (NK)	22	Pig	Canada	Chen et al., 2013	DRR018568

*NT, non-typeable; NK, detailed clinical phenotype not known.*

**FIGURE 1:**
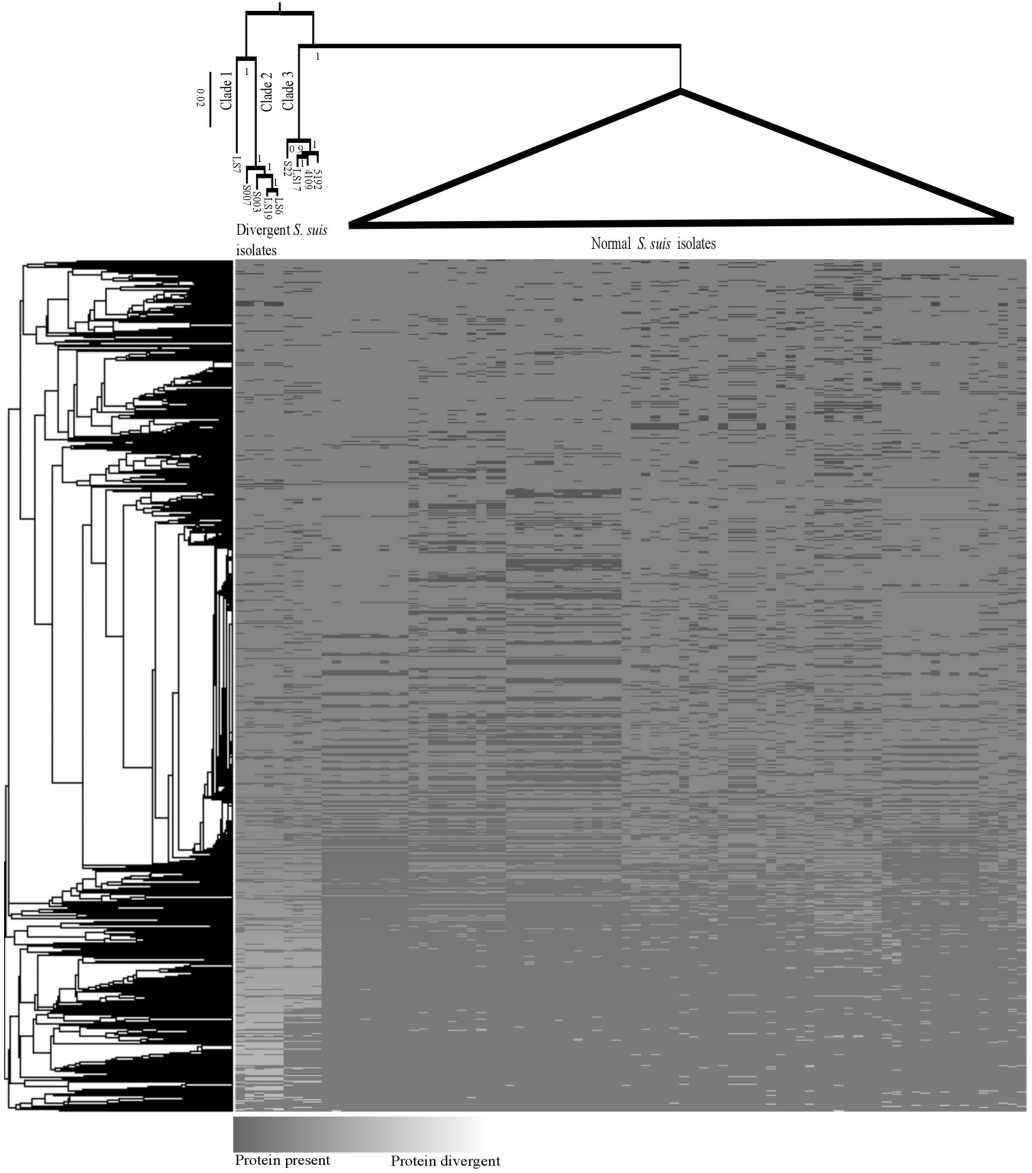
**Maximum likelihood phylogeny (top) of core genes for the divergent and normal *Streptococcus suis* isolates.** The branches with normal *S. suis* isolates have been collapsed. A heatmap showing the presence/divergence of proteins in the *S. suis* pan-genome and a cluster grouping those genes that co-occur in the same isolates (left).

**FIGURE 2:**
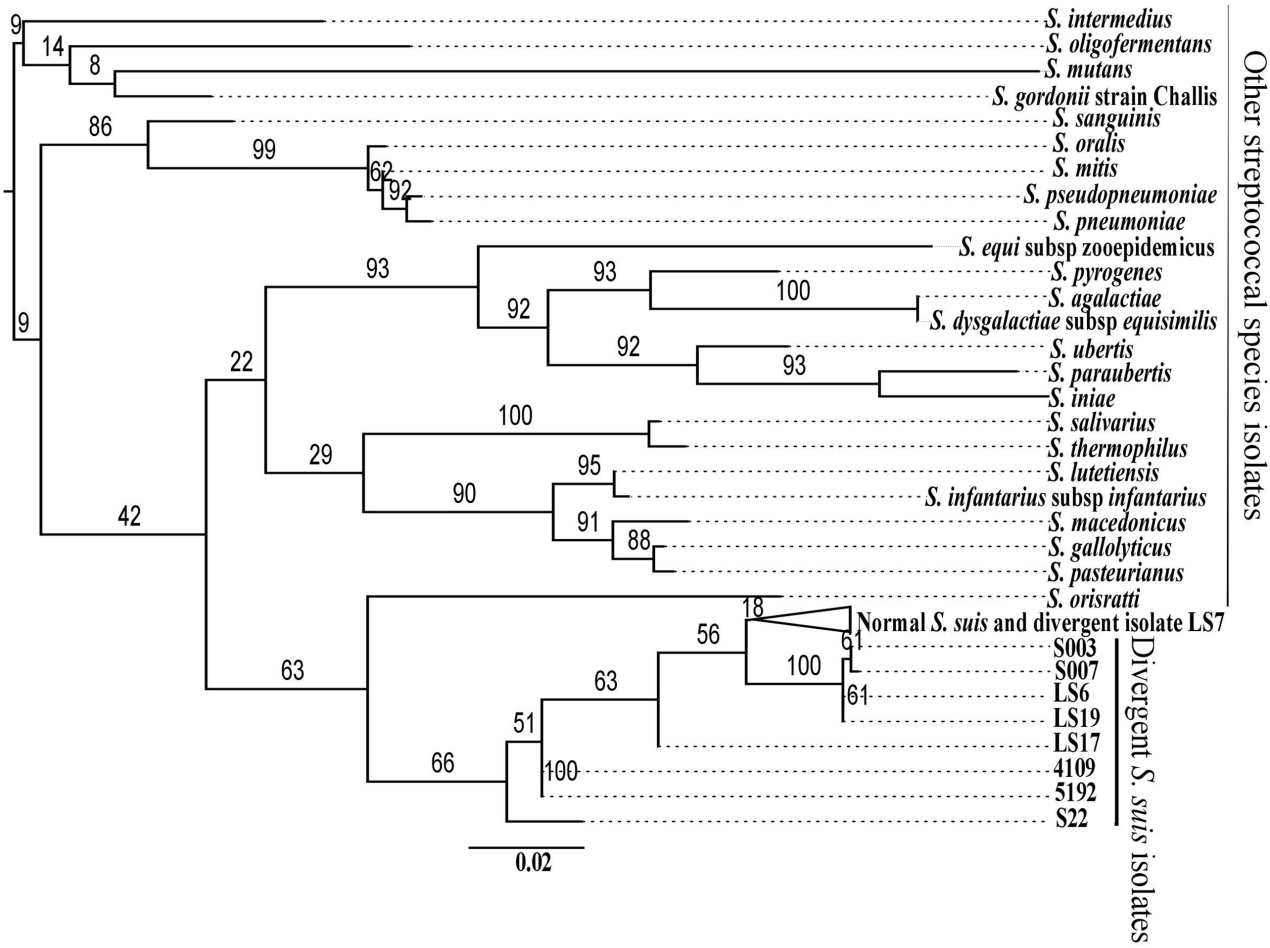
**Maximum likelihood phylogenetic reconstruction of the *16S rRNA* gene sequences of the divergent isolates with those from normal *S. suis* and representatives of the other 24 streptococcal species.** The branches with the 73 normal *S. suis* isolates and the divergent isolate LS7 have been collapsed.

**FIGURE 3:**
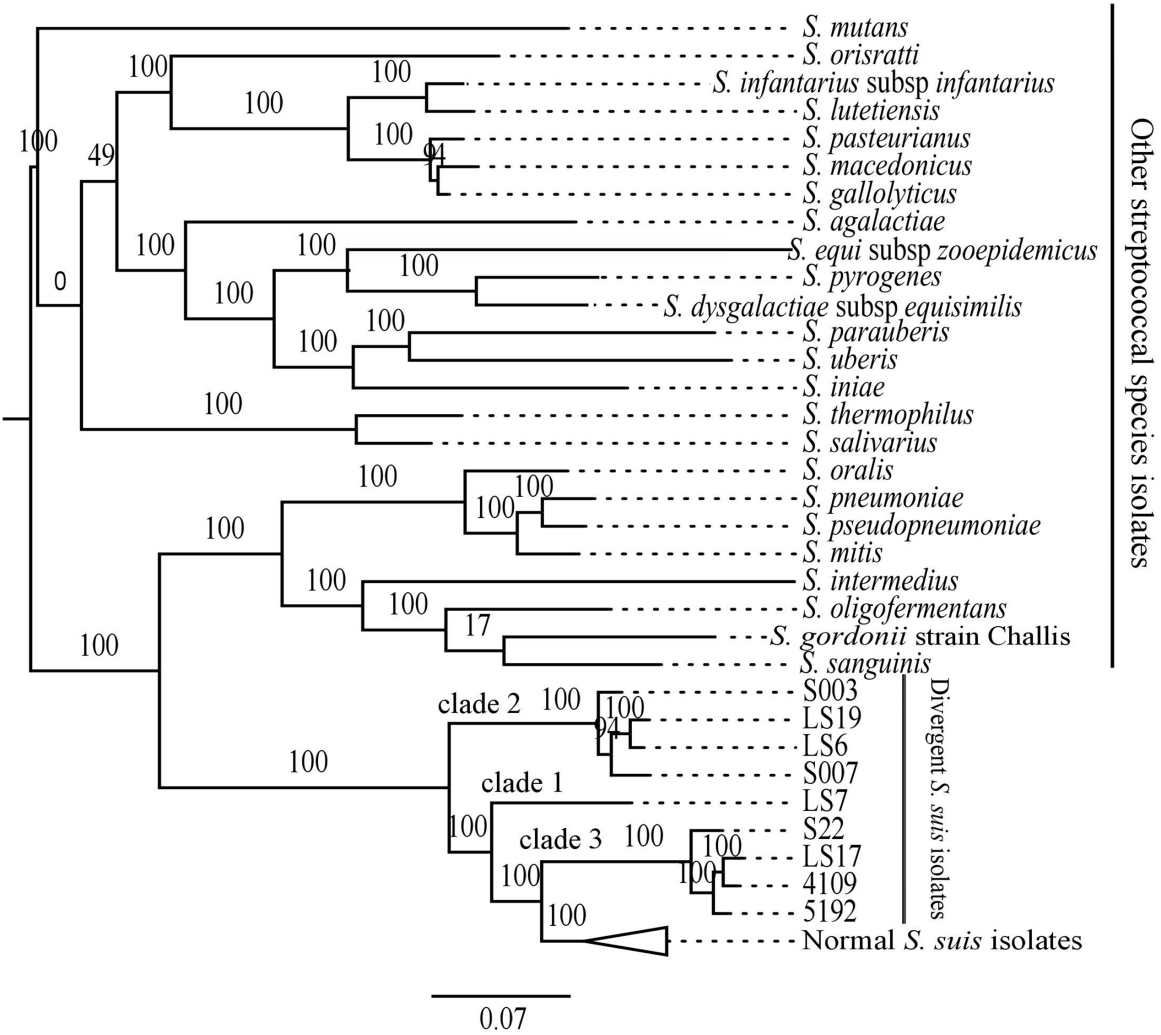
**Maximum likelihood phylogenetic comparison of the divergent isolates and representatives of the other 24 streptococcal species isolates, including 73 normal *S. suis*, based on shared subset of 132 genes.** The branches with the normal *S. suis* isolates have been collapsed.

**FIGURE 4:**
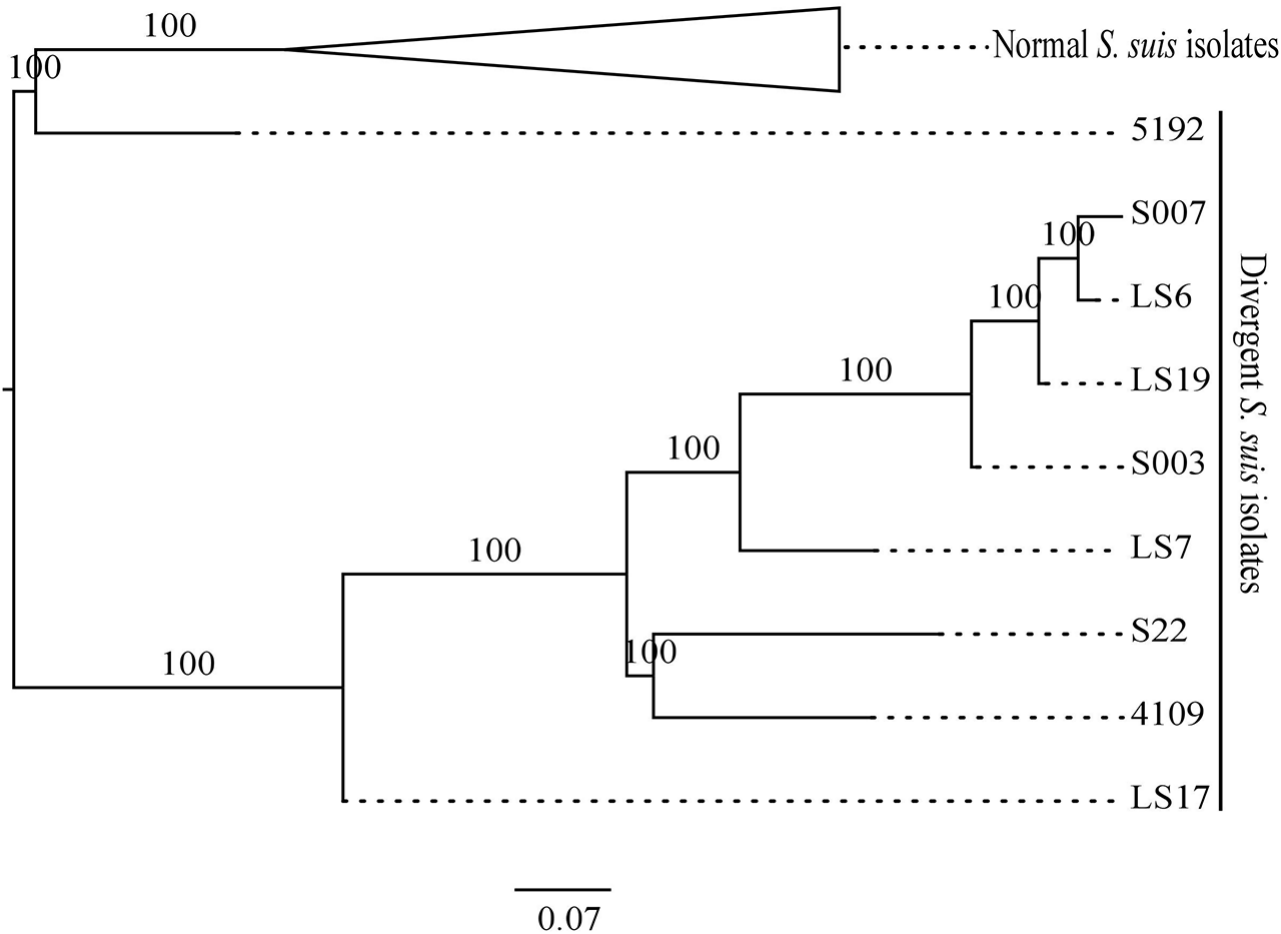
**Maximum likelihood phylogeny comparing the MLST allelic profiles of the divergent isolates with a selection of normal *S. suis* isolates.** The branches with the normal *S. suis* isolates have been collapsed.

A representative subset of 73 isolates (Supplementary Table S2) from the larger collection of 390 isolates of normal *S. suis*, was selected to make the phylogenetic trees shown in the figures (**Figures 1–4** and Supplementary Figure S2). This subset formed part of a previously reported large-scale genomic analysis of 390 isolates of *S. suis* (Weinert et al., 2015) and they are also listed in Supplementary Tables S1 and S2 of this study. Weinert et al. (2015) showed that the 390 isolates were distributed between five populations that were determined using Bayesian analysis of population structure (BAPS; Chen et al., 2013; Supplementary Table S1). For the subset of 73 normal *S. suis* isolates, we selected isolates from each of the five BAPS populations, ensuring representation of the same phenotypic characteristics as for the divergent isolates (Supplementary Table S2). For example, we selected representatives of clinical, non-clinical, and unknown disease phenotypes (Weinert et al., 2015). One representative of each serotype present in each BAPS group was included in the selection, with the exception of serotypes 4, 20, 22, and 26. Since these are the same serotypes as some of the divergent isolates (**Table 1**), all the genome sequences of normal *S. suis* with these four serotypes were selected. To represent non-typeable isolates, a maximum of five non-typeable normal *S. suis* isolates were selected from each of the BAPS groups, if present. Fifteen completed published genome sequences of *S. suis* were also included in this subset of 73 isolates (Supplementary Table S2). The genome sequences of these 73 normal *S. suis* isolates were compared with those from the six divergent isolates from the UK and the three divergent isolates from Canada (**Table 1**). Finally, one representative genome sequence from each of the other sequenced streptococcal species (*n* = 24) was included in the *16S rRNA* gene and streptococcal species core genome analysis (Supplementary Table S2).

### Comparison of Divergent *S. suis* Genomes with the Pan-genome of Normal *S. suis*

The pan-genome derived from sequences of all 390 normal *S. suis* isolates was defined by Weinert et al. (2015). Each homology group described therein represented distinct genes or proteins encoded by multiple isolates. The genomes of divergent isolates were compared with the normal *S. suis* by combining them into the pre-defined pan-genome of normal *S. suis* (Weinert et al., 2015). The coding sequences (CDSs) from all the divergent isolates were predicted using prodigal (Hyatt et al., 2010).

To find the homologous genes between the divergent *S. suis* and normal *S. suis* isolates, the reciprocal-best-blast method was used to compare the proteins from divergent isolates with those from the normal *S. suis*. This method provided 881 core or essential genes shared between the divergent isolates and normal *S. suis*. For these genes, the blast identities ranged from as low as 23% across 18% length of the protein. Such variation could be due to different rates of evolution in particular lineages and inter species recombination, which may lead to the wrong assignment of sequences as homologs. To avoid alignment errors and to infer a reliable phylogenetic relationship between the divergent isolates and normal *S. suis*, a stringent cut-off was used for inclusion of the proteins from divergent isolates into the pre-defined homology groups of *S. suis* (Weinert et al., 2015). To keep the sequence identity criteria consistent with our whole genome sequence study of normal *S. suis* isolates (Weinert et al., 2015), we selected proteins from divergent isolates that showed a minimum of 80% amino acid identity across 80% of the length of the protein when queried against the pre-defined *S. suis* pan-genome (Weinert et al., 2015) using BLASTp. These proteins were added into the respective *S. suis* homology groups. The homology groups which were present in all nine divergent and 390 normal *S. suis* isolates were considered conserved between divergent and normal *S. suis* isolates. The corresponding nucleotide sequences of proteins in conserved homology groups were aligned using MUSCLE (Edgar, 2004). The alignments of all these genes per isolate were concatenated and a phylogenetic comparison was performed using a generalized time reversible model (gtr) in FastTree with 1000 bootstrap repeats (Price et al., 2009). A heat map comparing the *S. suis* pan-genome with the divergent isolates’ pan-genome shared with *S. suis* was made by using a custom made R script. Any homology groups that were in less than 5% of isolates were excluded from display in the heat map.

### Homology Groups Specific to Divergent Isolates

The proteins of the divergent isolates which did not fit into the *S. suis* pan-genome were classified into new divergent-specific homology groups using OrthoMCL (Fischer et al., 2011). We used an MCL inflation parameter of 2.6, as was used for defining the homology groups in the *S. suis* pan-genome (Weinert et al., 2015) to define the maximum number of homology groups. All the MCL homology groups were manually checked in order to reduce the number of false positive sequences and duplicated protein sequences from the same isolate by checking the corresponding sequence alignments and alignment statistics generated using alistat (Eddy, 2005). Any protein which had a length less than 80% or greater than 120% of the modal length of all the proteins within the homology group was defined as being encoded by a pseudogene of the respective protein using custom Python scripts.

Once all the checks were completed, custom Python scripts were used to produce the MCL table for the altered homology groups. The normal and divergent isolates shared homology groups and the divergent-specific homology groups were used to define the core genome of the divergent isolates. The remaining homology groups contained the accessory genome for the divergent isolates.

### Individual Gene Analysis

The *16S rRNA* gene of the divergent isolates was compared with the *16S rRNA* genes of other streptococcus species (Supplementary Table S2). Individual housekeeping genes such as *recN, sodA*, and *cpn60* were used to characterize the relatedness of divergent isolates to normal *S. suis* isolates. The gene sequences of *16S rRNA, recN, sodA*, and *cpn60* from *S. suis* P1/7 were used to query against a database containing nucleotide sequences from all 390 normal *S. suis* isolates and nine divergent isolates in a BLASTn search.

Custom Python scripts were used to extract the nucleotide sequences of each gene from the genome sequence of each isolate based on the start and end co-ordinates identified by the BLAST output. For some *S. suis* isolates the complete gene sequences were not assembled so they could not be extracted. In such cases, the raw sequence reads were mapped against the gene sequence from *S. suis* P1/7 using Stampy (Lunter and Goodson, 2011) and the consensus sequence was extracted using SAMtools and BCFtools (Li et al., 2009). The *16S rRNA* gene sequences of published *Streptococcus* species isolates other than *S. suis* were obtained from NCBI. The nucleotide sequences were aligned using MUSCLE and a maximum likelihood phylogeny was constructed using RAxML, using the general time reversible (GTR/REV) model with the CAT approximation of rate heterogeneity (Stamatakis, 2006) and 100 non-parametric bootstrap replicates performed using the rapid algorithm (Stamatakis et al., 2008) and displayed using FigTree (n.d.)^1^.

### Multi-gene Analysis

To define further the relationship of divergent isolates with other species in the *Streptococcus* genus, a larger subset of proteins which showed at least 80% identity across 80% of the sequence length (as determined using BLASTp) from divergent and selected normal *S. suis* and representative genomes of the *Streptococcus* genus (Supplementary Table S2) was selected using a custom Perl script. This script concatenated the sequences from this subset of proteins to produce a core alignment. A phylogeny was constructed from this alignment using RAxML as above.

For comparison of divergent isolates with normal *S. suis* isolates based on the MLST scheme for *S. suis* (King et al., 2002), the published alleles for the MLST genes (*aroA, cpn60, dpr, gki, mutS, recA*, and *thrA*) recorded in the MLST database^2^ were downloaded for inclusion in the analysis. Initially, one allele for each gene was used as query to perform BLASTn against the nucleotide database of all nine divergent isolates and all 390 normal *S. suis* isolates in our collection. A custom Python script was used to filter out the nucleotide sequences of all alleles from the genome sequence of each isolate based on the nucleotide positions of each allele in the BLAST results, which were aligned using MUSCLE and a maximum likelihood phylogeny was drawn using RAxML as stated above.

### Recombination of Divergent Isolates with *S. suis* Species

To determine if the divergent isolates were recombining with normal *S. suis*, the nucleotide sequences of all the core genes from the divergent and the normal *S. suis* isolates were aligned using MUSCLE (Edgar, 2004). The phylogenetic comparison of each gene alignment was performed by FastTree as above. A custom R script was used to measure recombination between divergent and normal *S. suis* isolates. The script reported whether normal *S. suis* formed a monophyletic clade to the exclusion of divergent *S. suis*. For each gene, if the divergent isolates had not recombined with normal *S. suis* then they would form a monophyletic outgroup. If one or more of the divergent isolates had a normal *S. suis*-type gene, then the divergent isolates would be paraphyletic with normal *S. suis* and that gene would indicate a recent recombinant between the divergent and normal *S. suis*.

### Capsule Locus

The protein sequences of all capsule-related CDSs from all known *S. suis* serovars (serotypes 1–34 and serotype 1/2) were compared (BLASTp) against a database containing protein sequences of CDSs from all the divergent isolates. For each of the divergent isolates, the proteins that had a minimum of 80% identity across 80% of the length of a protein encoding a capsule gene were selected. Once the capsular genotype of each of the divergent isolates was known, an alignment comparison figure was generated against the capsular locus from the appropriate reference isolate using Easyfig (Sullivan et al., 2011)

### Virulence Genes in Divergent Isolates

The coding sequences of published virulence-associated proteins in *S. suis* (Fittipaldi et al., 2012) were obtained as Fasta sequences from NCBI. These sequences were used to perform a BLASTp search on a database containing protein CDSs from all divergent isolates. A virulence gene was only regarded as a homolog in divergent isolates if it showed at least 80% identity in protein sequence across 80% of the length of the protein.

## Results

Whole genome sequencing of a collection of clinical and non-clinical *S. suis* isolates from pigs in the UK identified a group of six isolates (S007, S003, LS17, LS19, LS6 and LS7; **Table 1**), which shared phenotypic and biochemical characteristics with normal *S. suis* but which had divergent genome sequences when compared with those of normal *S. suis*. Three other published genome sequences of *S. suis* (4109, 5192 and S22; **Table 1**; Chen et al., 2013) were also divergent. Here, we report the whole genome sequence analysis of these nine isolates in order to compare their genome diversity with that of normal *S. suis* and other representatives of the *Streptococcus* genus, listed in Supplementary Table S2.

### Comparison of Protein Sequences from Divergent versus Normal Isolates of *S. suis*

To investigate the similarity of proteins from divergent isolates with those from normal *S. suis*, we investigated the homology of proteins from divergent isolates against the pre-defined protein homology groups of a larger collection of 390 *S. suis* isolates (Weinert et al., 2015). Out of a total of 18997 (non-unique) proteins, 13505 proteins from the divergent isolates were identified as belonging to the pre-defined homology groups of the normal *S. suis* pan-genome (Weinert et al., 2015). The remaining 5492 proteins which did not fit into the *S. suis* pan-genome were put into 1244 new divergent isolate-specific homology groups, comprising 1089 genes and 155 pseudogenes, using OrthoMCL (Fischer et al., 2011).

A previously defined core genome of *S. suis* contained 793 genes (Weinert et al., 2015). However, when proteins from the divergent isolates, which matched with normal *S. suis* proteins to 80% identity across 80% of the length of the protein, were included, the genes shared between divergent and normal isolates was reduced to a core genome of only 397 genes. It is important to note that, since there is evidence for extensive recombination within *S. suis* (Weinert et al., 2015) and the *Streptococcus* genus in general (Hanage et al., 2009), the genome cannot be represented with one evolutionary history. We built a single core genome phylogeny to illustrate the differences between the divergent clades and the normal *S. suis* clade. There was a clear diversification of the nine divergent isolates into three clades separate from normal *S. suis* (**Figure 1**). A heatmap comparing the protein sequences of nine divergent isolates which showed at least 80% identity across 80% of length of the protein with the pan-genome of normal isolates of *S. suis* (**Figure 1**) showed that these nine isolates have more diverse genomes compared to the variability seen amongst the normal *S. suis* genomes. The proteins conserved in the divergent and normal *S. suis* isolates are represented at the bottom of the heatmap. The 397 core proteins of all the nine divergent and normal isolates of *S. suis* are also included in this region. The top-most region of the heatmap mainly represented isolate-specific protein sequences, which formed part of the accessory genome. Within the divergent clades, there was no grouping of isolates based on disease phenotype, country or serotype.

The most divergent clade, which branched off from the root of the tree, was composed of five divergent isolates. Within this group, clade 1 was represented by only one divergent isolate, LS7, which was highly divergent from all other isolates. The heatmap also showed LS7 as the most dissimilar isolate amongst all the divergent isolates compared to the normal *S. suis* isolates. The other four isolates (S007, S003, LS19, LS6) clustered together (clade 2). A third group of divergent isolates (clade 3) comprised the three published genomes from Canada (S22, 5192, 4109) along with LS17, and was more closely related to normal *S. suis*.

We found 914 genes in the core genome of the divergent isolates. There were 161,658 SNPs within the nine divergent isolates, a comparable number to the 178,979 SNPs found in the much larger collection of 390 normal *S. suis* isolates (Weinert et al., 2015) suggesting the diversity in *S. suis* has previously been considerably underestimated. The total assembly sizes of the divergent isolates ranged between 2.09 and 2.28 Mb which is similar to the assembly sizes of the 390 *S. suis* isolates (1.9–2.4 Mb) and the assembled genome sizes of other sequenced streptococcal species (1.64–2.43 Mb; Gao et al., 2014). The number of genes and pseudogenes in the divergent isolates ranged between 1964–2201 and 31–59, respectively (**Table 2**). The divergent isolates, as a group, had approximately sixty more genes compared to the group of isolates of normal *S. suis* with systemic, respiratory, and non-clinical phenotypes. This difference in the number of genes is calculated as a median of the total number of genes for each group. The divergent isolates from clinical cases had more genes compared to the clinical normal *S. suis* isolates but there was no difference in the number of genes in non-clinical divergent versus normal *S. suis*. Notably, the number of genes in the clinical and non-clinical divergent isolates was also similar (Supplementary Figure S1).

**Table 2:** The genome features of divergent isolates.

Divergent *S. suis* isolate ID	Assembly size (Mbp)	N50^∗^ (bp)	Number of genes	Number of pseudogenes
S007	2.15	18911	2108	54
S003	2.14	22250	2084	57
LS17	2.14	22031	2129	37
LS19	2.09	28411	2021	38
LS6	2.20	23839	2131	55
LS7	2.17	22353	1964	59
5192	2.11	86346	2013	31
4109	2.18	64745	2079	37
S22	2.28	35704	2201	38

*^∗^At least 50% of the assembly consists of contigs of the N50 size or greater.*

### Genomic Comparison of Divergent Isolates with Other Streptococcal Species

Comparison of proteins from divergent isolates with the pan-genome of normal *S. suis* showed that the divergent isolates were very different (**Figure 1**). To investigate whether the divergent isolates were more related to other streptococcal species than to *S. suis*, the nine divergent *S. suis* genomes were compared with 24 representative genomes from other species of *Streptococcus*, and to a subset of 73 normal *S. suis* genomes, selected as described earlier, using the sequence of the *16S rRNA* gene. Phylogenetic analysis showed that the divergent isolates were distributed in the *S. suis* clade and that the other streptococcal species branched off into a separate clade (**Figure 2**). Within the *S. suis* clade, eight of the nine divergent isolates were present on distant branches compared with the normal *S. suis* isolates, the exception being LS7, which was within the normal *S. suis* clade (**Figure 2**). The cluster with the published divergent isolates 4109 and 5192, and the branches with the published divergent isolate S22, and the divergent isolate LS17 were most distant from normal *S. suis*. Additionally, the percentage identity of the *16S rRNA* gene sequence from divergent isolates was compared against the *16S rRNA* gene sequence from P1/7. The *16S rRNA* gene sequences from all divergent isolates had more than 97% identity with that from P1/7, and an identity greater than 97% is considered to be the threshold for classification within the same bacterial species (Doolittle and Papke, 2006).

We compared the divergent isolates with other streptococcal species using protein-coding genes to give a comparison with the phylogenetic analysis based on the *16S rRNA* gene sequence. To achieve this, we identified common proteins which showed at least 80% protein sequence identity over at least 80% of their length among the nine divergent isolates, the representative group of 73 normal *S. suis* isolates, and representative isolates of 24 other streptococcal species (total of 106 isolates; Supplementary Table S2). This defined 132 genes as present in all the isolates considered. The phylogeny based on this larger subset of 132 shared genes showed that the divergent *S. suis* may have emerged from an ancestral population prior to normal *S. suis* (**Figure 3**). The divergent isolates formed a basal group to normal *S. suis*, placing the divergent isolates in three clades. Clade 1 (LS7) and clade 2 (S007, S003, LS19, LS6) were most distant from normal *S. suis*, and clade 3 (S22, LS17, 4109, 5192) was the closest. This clustering of divergent isolates was similar to that identified in the pan-genome phylogeny (**Figure 1**). An obvious incongruence between the *16S rRNA* gene and the shared gene phylogenies was the positioning of clade 1 (**Figure 3**), which in the *16S rRNA* gene phylogeny positioned LS7 within the normal *S. suis* clade.

### Comparison of Housekeeping Genes and MLST between Divergent and Normal *S. suis*

In the current study, the phylogenetic analysis based on the *recN, sodA*, and *cpn60* gene sequences showed inconsistent results (Supplementary Figure S2). Only the phylogeny based on *recN* differentiated these isolates as divergent from normal *S. suis* (Supplementary Figure S2A). The phylogeny based on *sodA* placed the divergent isolate LS7 within normal *S. suis* (Supplementary Figure S2B). Based on *recN* and *sodA* phylogenies, the three published divergent isolates, 5192, S22, and 4109 (serotypes 20, 22, and 26, respectively) clustered with the divergent non-typeable isolate LS17. Notably, this group of four divergent isolates appeared as part of the normal *S. suis* cluster based on *cpn60* gene sequence (Supplementary Figure S2C).

The published MLST scheme in *S. suis* utilizes variation in the housekeeping genes *aroA, cpn60, dpr, gki, mutS, recA*, and *thrA* (King et al., 2002) to help define the population structure (reviewed in Desjardins et al., 2014). Currently, there are 618 sequence types (STs) and 1373 allelic profiles in the MLST database^2^. Phylogenetic analysis of the divergent isolates and the 73 selected normal *S. suis* isolates using concatenated MLST allelic sequences distinguished between the divergent and normal *S. suis* isolates (**Figure 4**). MLST-based phylogeny distributed the divergent isolates into two clades. Within these clades, the published divergent isolate 5192 was present on a separate branch, closer to the normal *S. suis* isolates. The remaining eight divergent isolates were more distant from normal *S. suis*. The divergent isolate LS17 was present on a node ancestral to the seven divergent isolates (i.e. 4109, S22, LS7, S003, LS19, LS6 and S007).

It is important to note that there are mismatches in the genomic DNA sequence of the divergent isolates at the positions at which the published primers for the MLST alleles would anneal (King et al., 2002), as shown in the Supplementary Table S5. This means that the published MLST scheme would be unlikely to identify the divergent isolates.

Overall, the individual gene analyses showed mixed results. Phylogenies based on *16S rRNA, sodA*, and *cpn60* genes could not discriminate all of the divergent isolates from normal *S. suis*, indicating that there may be recent gene exchange or less likely strong convergent evolution between the divergent and normal isolates.

### Recombination with Normal *S. suis* Population

We investigated how many of the individual genes from 397 conserved genes displayed an anomalous phylogeny with respect to distinguishing between the divergent and normal *S. suis* populations. We constructed phylogenies of each of the 397 conserved genes from the nine divergent genomes and the 390 normal *S. suis* genomes. Of 397 conserved genes, 148 exhibited phylogenies that supported a monophyletic grouping of the divergent isolates (Supplementary Table S3). For the other genes, at least one of the divergent isolates grouped within the main normal *S. suis* clade indicating recombination between them.

### Capsule Locus in Divergent Isolates

The divergent isolates belonged to serotypes 4, 20, 22 and 26, or were non-typeable (**Table 1**). This suggests either that these non-typeable *S. suis* have novel capsules, or that they lack the capsule genes. The capsule region of the divergent isolates was identified by performing BLASTp searches of the capsule protein sequences of all *S. suis* serotypes, including the recently reported divergent serotypes 20, 22 and 26, now classified as *S. parasuis* sp. nov (Nomoto et al., 2015), and serotype 33 (Le et al., 2013). The non-*S. suis* serotypes 32 and 34 which are now considered to be more closely related to *S. orisratti* (Brousseau et al., 2001; Hill et al., 2005) were also included and compared against a database consisting of all proteins predicted to be encoded by the divergent isolates.

**FIGURE 5:**
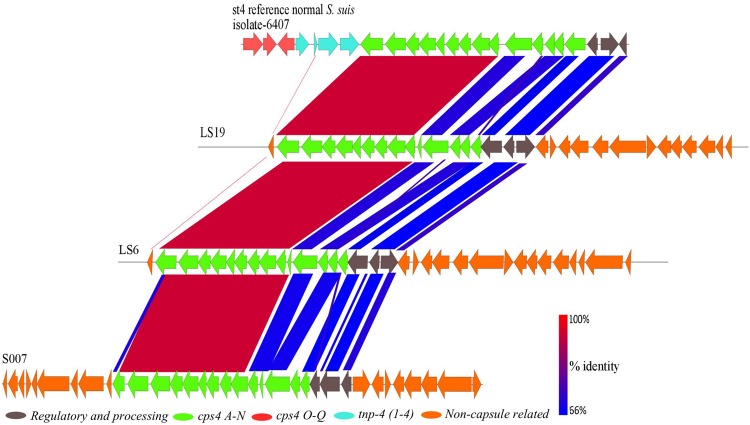
**A BLASTn alignment of the 26.5 kbp capsular region of the normal *S. suis* serotype 4 reference isolate (top) with the divergent serotype 4 isolates, constructed using EasyFig**.

Two divergent isolates, S007 and LS19, had been classically serotyped as serotype 4 and the divergent isolate LS6 as non-typeable. BLASTp comparisons with capsule genes of the serotype 4 reference isolate (6407) showed that these three divergent isolates all possessed a serotype 4 capsule region (**Figure 5**). The serotype 4 capsule locus from the reference isolate is composed of 17 genes (*cps4A–Q*), six of which are serotype 4-specific (*cps4H–M*), with the rest being shared with one or more of the other *S. suis* serotypes (Okura et al., 2013). Genes that were serotype 4-specific and *cps4F, G* and *N*, in the normal *S. suis* serotype 4 reference isolate, were present in these three divergent isolates, with 95–98% identity at the amino acid level. The predicted proteins encoded by *cps4A–E* and regulatory and processing genes, in the normal *S. suis* serotype 4 reference isolate, had less than 85% amino acid sequence identity with their homologs in these three divergent isolates. Two predicted proteins with less than 30% identity encoded by *cps4O* and *cps4Q* were located on different contigs in the genomes of these isolates and a predicted protein encoded by *cps4P*, in the normal *S. suis* serotype 4 reference isolate, was not identified. Notably, none of the three serotype 4 divergent isolates had any capsular genes with 100% identity at the protein level to the predicted proteins encoded by the serotype 4 capsule genes of the reference isolate. Interestingly, these divergent isolates also possessed predicted proteins with similarity to conserved predicted proteins encoded in serotypes 22, 26, 32, 34, by genes dispersed in their genomes. The conserved genes in these serotypes were *cps22O–R*, Y and Z, *cps26R–U, cps32M–P, cps34O, P, R*, and *S*.

The divergent clinical isolate S003 had been characterized as serotype 22. Whole genome analysis showed that S003 did not contain a serotype 22 capsule cluster or a capsule cluster of any other serotype. The two non-clinical divergent isolates LS7 and LS17, which were non-typeable, did not possess a capsule cluster either. In these three isolates, the capsule genes were scattered over different contigs across the genome. The majority of capsule genes found in these isolates were similar to capsular genes common to the serotypes 22, 26, 32, and 34.

The published divergent isolate, S22, is the serotype 22 reference isolate. There are 26 genes in the S22 capsule cluster and 16 genes, from *cps22F*–*N*, and *cps22T*–*Z*, that are specific to the serotype 22 capsular region (Okura et al., 2013). Another published divergent isolate, 4109, is the reference isolate for serotype 26. This isolate has 23 genes in the capsule cluster, 10 of which (*cps26F, G, I, J, L–P, T*) define this serotype (Okura et al., 2013). Finally the published divergent isolate 5192 is serotype 20. This serotype has 18 genes in its capsule cluster with 7 (*cps20G–M*) defining the serotype (Okura et al., 2013).

### *S. suis* Virulence Gene Homologs in Divergent Isolates

Given that some of the divergent isolates are capable of causing disease, or at least have been cultured from systemic sites, we searched for the presence of genes, which have been associated with virulence (Fittipaldi et al., 2012) in the genomes of the divergent isolates. Out of a total of 70 virulence genes investigated, we found that on average the divergent isolates had 40 genes homologous to the genes in normal *S. suis*. S22 had the most (46), whereas LSS7 contained the fewest (33) homologs of *S. suis* virulence genes. There was no difference in prevalence of these genes between clinical and non-clinical divergent isolates. The distribution of these genes amongst the divergent isolates is given in Supplementary Table S4. Twenty-four out of 70 virulence genes were absent from all nine divergent isolates. These included the genes encoding the muramidase-released precursor surface protein (*mrp*) and the large variant extracellular factor (*ef*). Twenty-nine virulence-associated genes were present in all nine divergent isolates. Among these were the genes encoding the ferric uptake regulator family protein (*fur*) and the fibronectin fibrinogen-binding protein (*fbpS*).

## Discussion

A group of nine isolates that were classified by laboratory methods as *S. suis* by their typical phenotypic characteristics were divergent from normal *S. suis* at the level of their whole genome sequences. First, a similar amount of diversity exists within these nine isolates as within all the genomes of normal *S. suis* isolates combined. Second, considerable differences in gene content were found in both the core and accessory genomes of these divergent isolates when compared with the normal *S. suis* isolates. Third, no genomic differences were detected between clinical and non-clinical divergent isolates (such as presence of virulence factors or difference in genome size), in direct contrast to normal *S. suis* (see Weinert et al., 2015) although this observation should be treated with caution as the sample size of the divergent isolates is small (*n* = 9). Many genes previously associated with virulence, like *mrp* and *ef*, in normal *S. suis* were absent from the divergent isolates. Finally, phylogenetic analysis based on 397 conserved genes (**Figure 1**) and the sequences of individual housekeeping genes such as *recN* (Supplementary Figure S2A), and those used in MLST (**Figure 4**), showed that the divergent isolates were only distantly related to normal *S. suis*. Indeed, the existing MLST primers for profiling of *S. suis* isolates would not be usable to type the divergent isolates. These points taken together could support the identification of these divergent isolates as a separate species from normal *S. suis* (as inferred by Nomoto et al., 2015), but we suggest that this re-classification would be premature.

At the whole genome sequence level, the divergent isolates showed a number of similarities to normal *S. suis*. The distribution of divergent *S. suis* isolates in the *16S rRNA* phylogeny (**Figure 2**) and the variation based on the larger subset of 132 genes shared between the divergent isolates and members of other streptococcal species (**Figure 3**) confirmed that the divergent isolates were more closely related to normal *S. suis* than to other streptococcal species. In individual gene trees, the groupings within the divergent clades were inconsistent and divergent *S. suis* grouped with normal *S. suis* in some analyses [for example, in phylogenies based on *16S rRNA, sodA*, and *cpn60* (**Figure 2** and Supplementary Figures S2B,C)]. This indicates recent recombination events between divergent and normal *S. suis* isolates. In addition, *16S rRNA* gene sequences in the divergent isolates were more than 97% identical to the *16S rRNA* gene sequence in normal *S. suis* isolate P1/7 (Doolittle and Papke, 2006), which suggests that they should be considered *S. suis* as opposed to a new species classified by Nomoto et al. (2015). The divergent isolates possessed 40 homologs of 70 virulence-associated genes associated with normal *S. suis* (Fittipaldi et al., 2012). Furthermore, two of the divergent isolates had a normal *S. suis* serotype 4 capsule (**Figure 5**), suggesting that these have either recombined with normal *S. suis* or that serotype 4 was present in the common ancestor of divergent and normal *S. suis*. Taken together, this calls into question the identification of the divergent isolates as a separate and distinct species. The species assignment is further hampered by the paraphyletic nature of divergent and normal *S. suis* (see **Figures 2–4**). This suggests that if the divergent isolates were to be classified as a new species separate from normal *S. suis*, three new species would need to be defined, one for each of the three clades. We are therefore clear that, on balance, these isolates should remain classified as divergent *S. suis*.

Our study highlights that the use of individual gene markers to type normal *S. suis* can be flawed. Phylogenetic comparisons based on the *16S rRNA, recN, sodA*, and *cpn60* gene sequences, which are used as molecular markers for speciation of normal *S. suis*, showed that relying on individual genes does not offer sufficient resolution to discriminate all of the divergent isolates from normal *S. suis*. In our study, using *recN* in isolation would lead to the view that the divergent isolates were distantly related to normal *S. suis* whereas using the most studied *16S rRNA, sodA*, and *cpn60* genes (Doolittle and Papke, 2006) does not discriminate all the divergent isolates from normal *S. suis*. This suggests that the divergent isolates (and perhaps normal *S. suis* isolates in general) can only be identified and reliably classified using whole genome sequencing.

## Conclusion

A group of divergent *S. suis* isolates, which were isolated from pigs and showed clinical and phenotypic properties of *S. suis*, had considerable diversity in their core and accessory genomes when compared to most *S. suis* isolates. Notably, the number of *S. suis* core genes reduced dramatically when analysis of the divergent isolates was included (397 versus 793; **Figure 1**). Phylogenetic analysis of the conserved *16S rRNA* gene sequence and a larger sub-set of 132 shared genes showed that the divergent isolates were closer to *S. suis* than to the other streptococcal species tested (**Figures 2** and **3**). Comparisons based on gene sequences of individual housekeeping genes including *16S rRNA, recN, sodA*, and *cpn60* could not discriminate all of the divergent isolates from normal *S. suis* (**Figure 2** and Supplementary Figure S2). The diversity seen in the MLST profile classified all these divergent isolates as distant from normal *S. suis* (**Figure 4**). For more variable genomic regions like the capsule locus, some divergent isolates showed evidence of recombination with normal *S. suis* whilst others lacked a classic capsular region (**Figure 5**). The recombination analysis based on 397 conserved genes between divergent isolates and normal *S. suis* indicated that recent recombination events had occurred in 249 genes. Phylogenetic analysis using whole genome sequences showed that the divergent isolates represent a basal population of the normal *S. suis* clade. The study of the divergent genomes, therefore, expands the diversity that had previously been found within *S. suis*. This diversity could lead to the evolution of enhanced phenotypes, for example, for virulence or antimicrobial resistance. Therefore, further extensive analysis of the whole genomes of larger populations of *S. suis* isolates is indicated, which will allow better understanding of the diversity and ecology of this significant zoonotic pathogen.

## Author Contributions

DM, AT, AR, PL, BW conceived the study; AB, JW, SP, AT produced the data; AB, LW, KH, RC analyzed the data; AB, LW, SP, KH, RC, MH, AT, DM wrote the paper.

## Conflict of Interest Statement

The authors declare that the research was conducted in the absence of any commercial or financial relationships that could be construed as a potential conflict of interest.

## Footnotes

1 http://tree.bio.ed.ac.uk/software/figtree

2 http://ssuis.mlst.net
