# Supplementary figures and images for: Whole genome investigation of a divergent clade of the pathogen Streptococcus suis
Source: Front Microbiol. 2015 Nov 4;6:1191. doi: 10.3389/fmicb.2015.01191 (PMC4631834; doi:10.3389/fmicb.2015.01191)

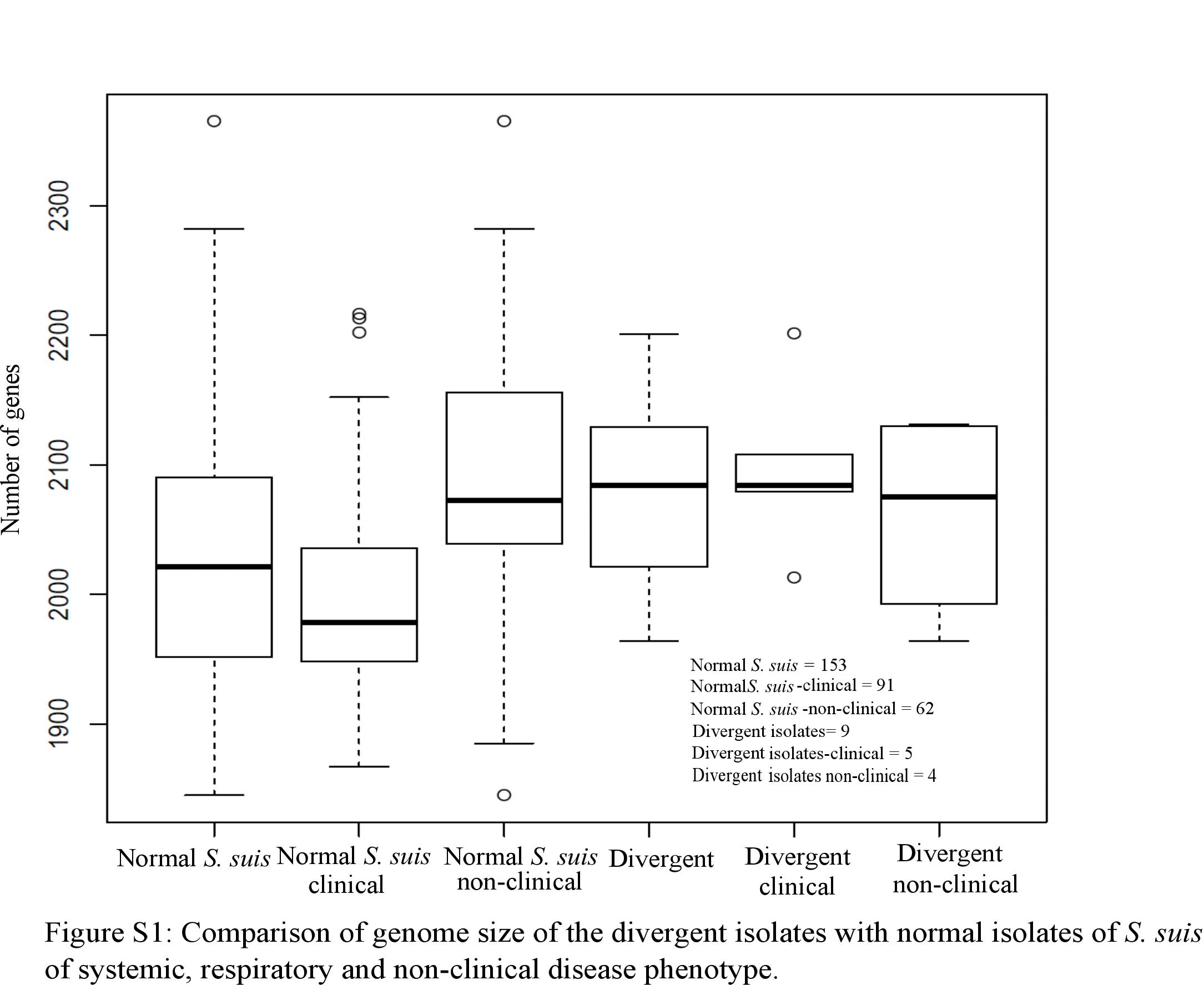

Supplement: Supplementary file 2 [file Image_1.JPEG]
