# Supplementary material for: Whole genome investigation of a divergent clade of the pathogen Streptococcus suis
Source: Front Microbiol. 2015 Nov 4;6:1191. doi: 10.3389/fmicb.2015.01191 (PMC4631834; doi:10.3389/fmicb.2015.01191)
Supplement: Supplementary file 3 [file Image_2.PDF]

A. *recN*

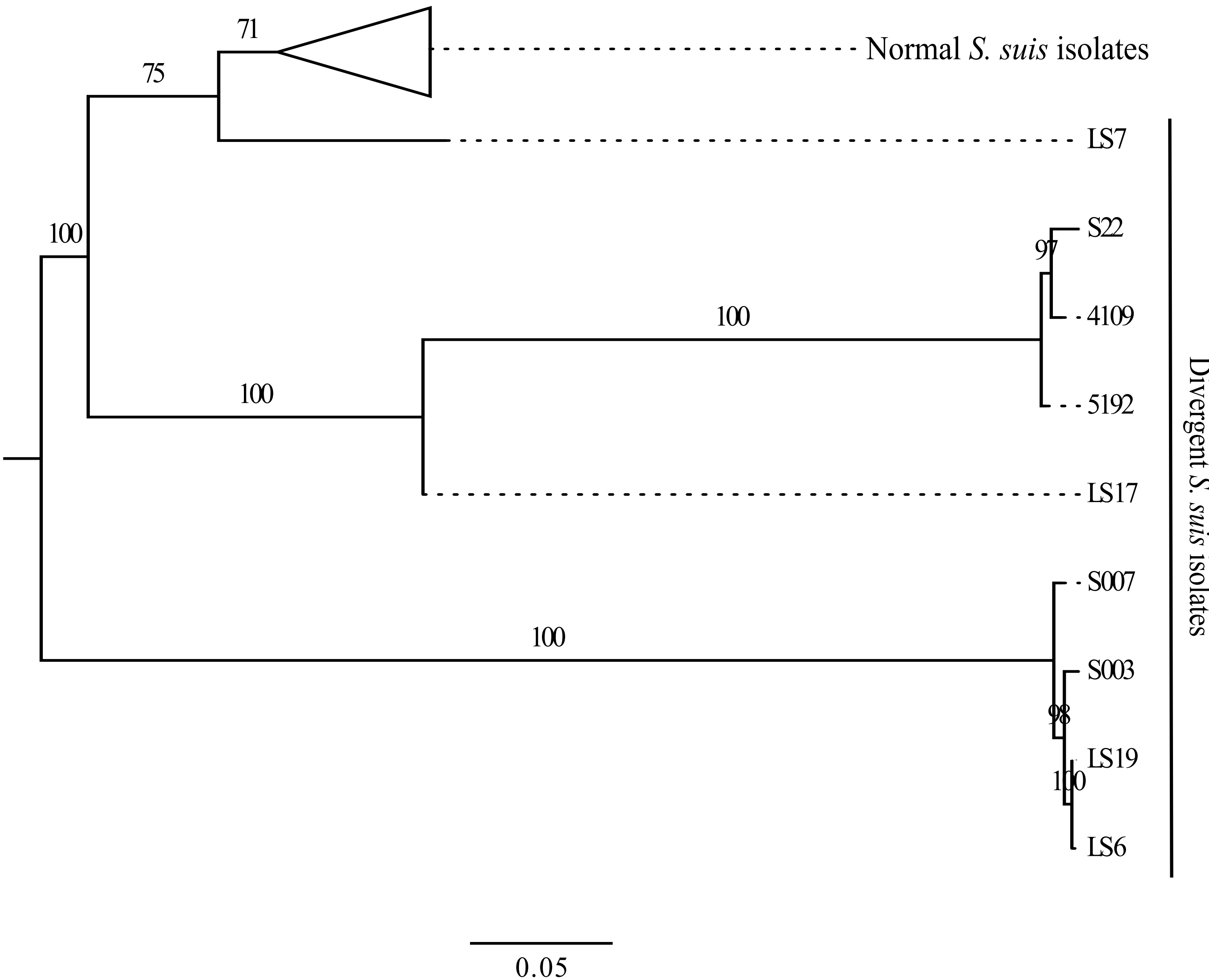
71
75
100
100
100
100
97
98
100
Normal S. suis isolates
LS7
S22
4109
5192
LS17
S007
S003
LS19
LS6
Divergent S. suis isolates
0.05

B. *sodA*

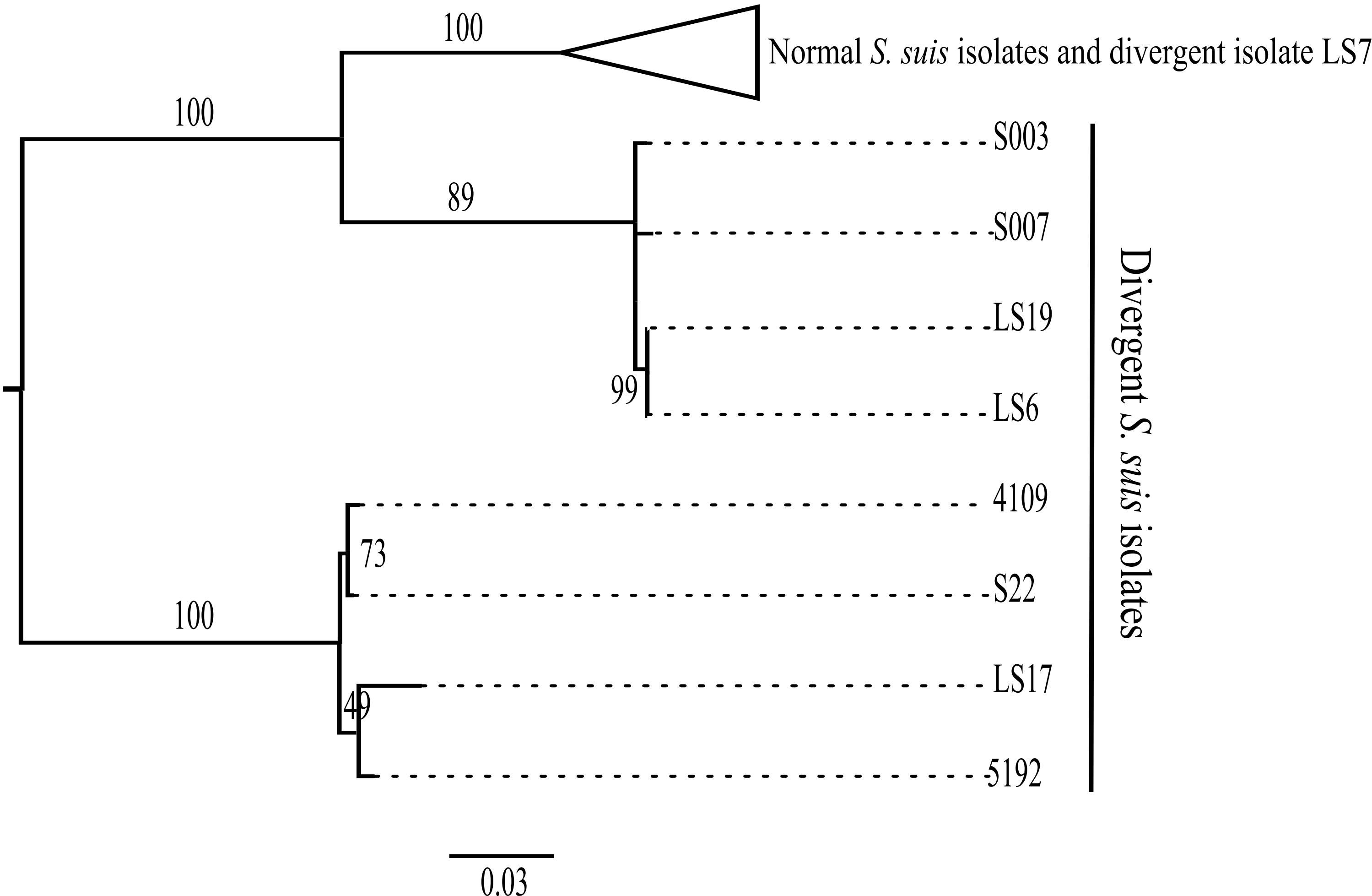
100
Normal S. suis isolates and divergent isolate LS7
100
S003
89
S007
LS19
99
LS6
4109
73
S22
100
LS17
49
5192
Divergent S. suis isolates
0.03

C. *cpn60*

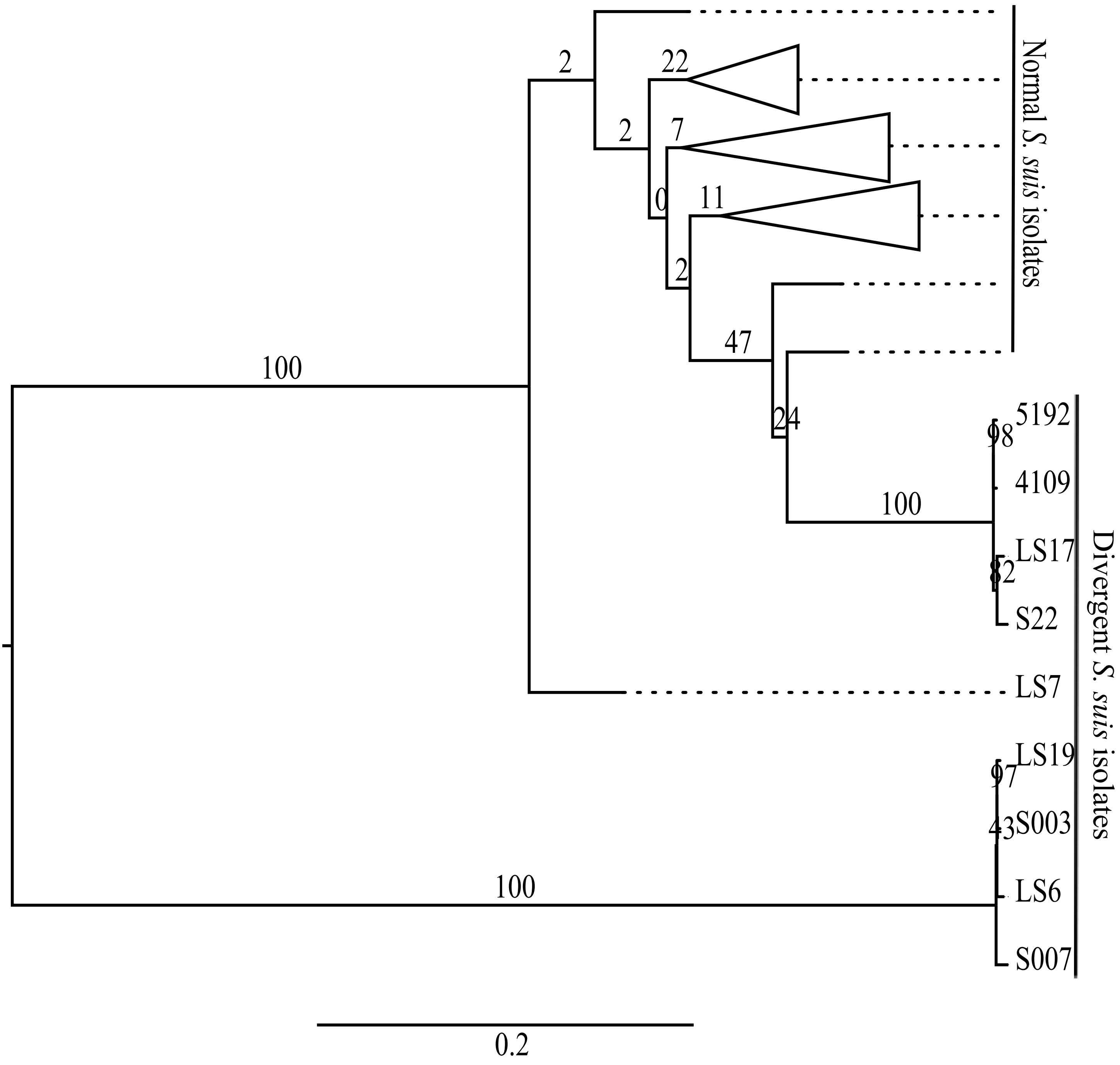

Figure S2: Phylogenetic comparison of the divergent *S. suis* isolates with selected normal *S. suis* isolates for (A) *recN*, (B) *sodA* and (C) *cpn60* gene sequences. The branches with the normal *S. suis* isolates have been collapsed.
